# Development of a 2-D Array Ultrasonic Transducer for 3-D Imaging of Objects Immersed in Water

**DOI:** 10.3390/s21103501

**Published:** 2021-05-18

**Authors:** Estevão Patricio Rodrigues, Timoteo Francisco de Oliveira, Marcelo Yassunori Matuda, Flávio Buiochi

**Affiliations:** Department of Mechatronic Engineering, Engineering School at the University of São Paulo, São Paulo 05508-010, Brazil; tim@usp.br (T.F.d.O.); marcelo.matuda@usp.br (M.Y.M.); fbuiochi@usp.br (F.B.)

**Keywords:** 2-D array, ultrasonic transducer, 1–3 piezocomposite, underwater, 3-D sonar, 3-D acoustical image

## Abstract

Most works that address 2-D array ultrasonic transducers for underwater applications are about the geometry aspects of the array and beamforming techniques to make 3-D images. They look for techniques to reduce the number of elements from wide apertures, maintaining the side lobes and the grating lobes at acceptable levels, but not many details about the materials and fabrication processes are described. To overcome these gaps, this paper presents in detail the development of a 2-D array ultrasonic transducer prototype that can individually emit and receive ultrasonic pulses to make 3-D images of immersed reflectors within a volume of interest (VOI). It consists of a 4 × 4 matrix ultrasonic transducer with a central frequency of 480 kHz. Each element is a 5 mm sided square cut into a 1–3 piezocomposite. The center-to-center distance of two contiguous elements (pitch) was chosen to be greater than half wavelength, to increase the amplitude of emission and reception of signals with larger elements. Artifacts generated by grating lobes were avoided by restricting the field of view in the azimuth and elevation directions within 40° × 40° and applying the sign coherence factor (SCF) filter. Two types of backing layer materials were tested, one with air and another made of epoxy resin, on the transducers called T1 and T2, respectively. The pulse echoes measured with T1 had 2.6 dB higher amplitude than those measured with T2, and the bandwidths were 54% and 50% @ −6 dB, respectively, exciting the element with a single rectangular negative pulse. The 3-D images obtained with full matrix capture (FMC) data sets acquired of objects from 0.2 to 1.15 m motivate the development of a 2-D array transducer with more elements, to increase the angular resolution and the range.

## 1. Introduction

A 2-D array ultrasonic transducer can be used to generate 3-D images of underwater objects up to several meters away, even if they are immersed in water with low or no visibility conditions, which is not possible with underwater optical cameras [1,2,3,4]. This capability makes it attractive for underwater purposes, such as gas and mineral extraction, finding archaeological artifacts and shipwrecks, mine hunting, navigation of divers, and autonomous underwater vehicles (AUVs).

Also called a planar array [2], a 2-D array ultrasonic transducer consists of a grid of individual piezoelectric elements, which are controlled to emit and receive signals. If the elements are driven at different instants of time, with previously calculated delays, a focused or defocused [1] wavefront is emitted.

The focused emission increases the concentration of acoustical energy in a given direction of interest, whereas it suppresses the echoes from the other directions. This improves the sensitivity and the contrast of images. However, only a beam is emitted at a time, and many beams are necessary to make a 3-D image, so the focused emission may not be suitable for underwater images in real-time, because the objects are too far from the sonar, more than usual in the non-destructive evaluation and medical ultrasound images. The time of flight of the echoes is limited by the speed of sound in water (1500 m/s). So, for example, to make a 3-D image frame from objects up to a meter away, within a VOI with 40° in azimuthal and elevation direction at steps of 1°, 1681 emissions of beams (considering only deflections or a fixed focal distance) are required to sample the frame and it would take at least 2.24 s.

On the other hand, the frame rate can be increased if the 2-D array transducer emits a defocused beam to the region with a single emission. The defocused emission is performed by driving the central elements before the peripherals, with delays previously calculated [1], so the backscattered echoes are sampled in parallel and coherently summed to form post-processed beams (delay-and-sum beamforming) [2,3,5]. Since a single emission is necessary to sample an entire region, imaging the same VOI is expected to be 1680 times faster. Furthermore, one can improve the images contrast by adjusting electronically the defocusing with delays, opening or closing the field of view [1]. Alternatively, a simpler 3-D sonar system can be developed by using a single omnidirectional transducer to irradiate the medium, such as a projector, and a 2-D array transducer, such as a bistatic sonar [2,3]. This simplifies the electronics because only a powerful transducer is required to irradiate the medium, but the field of view is not electronically adjustable.

There are not many works describing in detail the design and construction of 2-D array transducers for underwater purposes, with non-destructive testing (NDT) and medical applications being the most commonly found [6,7,8,9,10,11,12,13]. There is thus a lack of information about the type of piezoceramics, matching layer, backing layer, and construction process. Therefore, these key features must be researched for the development of a 2-D array ultrasonic transducer capable of three-dimensional underwater imaging. The most accessible features found directly or indirectly (obtained with equations) are the quantity and the size of the elements, the pitch, the aperture size, the central frequency, and the field of view.

A prototype of 3-D real-time image sonar composed of a spherical transmitter and a 48 × 48 planar array receiver is presented in [14]. The transmitter emits a gated sinusoid with a frequency of 300 kHz and a length of approximately 26 μs, resulting in a range resolution of 2 cm, considering the propagation velocity in water to be 1500 m/s (the range resolution can be defined as one-half the pulse length [15]). The grating lobes were avoided by making the pitch equal to λ/2. The field of view is 50° × 50° and the angular resolution is 2.1°. A 3D image of a wooden cube was taken from about 10 m away from the array.

A multi-beam sonar system consisting of a continuous wave emitter (emitting a 200 μs signal at 100 kHz) and a 2-D uniform planar array is presented in [5]. The center-to-center distance between the elements is equal to 2 λ (undersampled array) in order to increase the aperture of the array without changing the number of elements. The wider the aperture, the narrower the main lobe, and the better the angular/lateral resolution. The grating lobe effect is avoided by limiting the field of view to 30° × 30°. The range resolution is 0.384 m and the operational distance is 5 m to 40 m.

In [1], the development of a 3-D sonar intended for volumetric images at deep-sea waters is presented. It consists of a 32 × 32 array of 1–3 piezocomposite elements, which operates at the central frequency of 1 MHz. The center-to-center distance between two contiguous elements (pitch) is equal to 2 λ, resulting in a square aperture with sides of 96 mm. The electronically controlled defocused emission allows producing a field of view of 35° × 35° @ −3 dB.

A hypothesized receiver planar array for a 3-D sonar imaging system is designed and simulated in [2]. It consists of a 2-D array that operates at 600 kHz and 1.2 MHz with 25% of bandwidth. The aperture has 250 mm sides and the center-to-center distance between two adjacent elements is fixed at 2.5 mm. Operating at 600 kHz, the pitch is λ and the field of view is 52° × 52°. When it operates at 1.2 MHz, the pitch is 2 λ and the field of view is 26° × 26°. To reduce the costs and the complexity of the electronic, the 100 × 100 element array was placed inside a circular aperture and also optimally thinned up to become a 584 element planar sparse array. Simulated results of three-dimensional images of a cylinder 1 m away from the 2-D array were presented.

The development of a portable bistatic 3-D sonar system for mine reconnaissance is presented in [16]. It consists of a 2-D sparse array receiver that has 121 elements of 1 mm × 1 mm, with a central frequency of 2 MHz and a 667 kHz bandwidth, and the field of view is 20° × 20°. An experimental volume image of a cinder block 3 m distant from the 2-D array was presented.

A sparse array can be used to produce a transducer with a wide aperture, which provides high lateral resolution. On the other hand, the side lobes are increased and the signal-to-noise ratio is decreased, when compared to full populated array transducers [2,3]. The sparse array can be obtained from a dense array (arrays with a pitch limited to half of a wavelength) by randomly thinning its elements [2,3]. When the sparse array is obtained by removing elements with sides equals to λ/2 (the element size is limited by the pitch distance) the grating lobes can be suppressed by the aperiodicity of the positions of the elements. Although, when the elements are greater than λ/2 as in the transducer developed in [2], the grating lobes are not suppressed even with aperiodicity of the elements, requiring limiting the deflection of the beams emitted and received to avoid artifacts in the images.

A 3-D underwater imaging system composed of a sparse planar array with 375 elements, obtained by thinning a dense array of 100 × 100 elements using the simulated annealing algorithm, was designed in [17]. The pitch is equal to 2.5 mm (λ/2). The center frequency of the carrier is 300 kHz, and the sampling frequency is 900 kHz. The angular resolution @−3 dB is 1.28°, the range resolution is 2 cm and the field of view is 60° × 60°. Tests of 3D images included a bicycle 3 m away from the array and the bottom of the seawater was detected up to 40 m.

As an alternative to the traditional square arrays, which need a large number of elements to maintain a high azimuthal and elevation resolution in the images, a segmented annular array (SAA) for NDT applications was designed, fabricated, and characterized in [6]. As SAA transducers have spatial diversity and less periodicity than traditional 2-D square array transducers, images with equivalent quality can be obtained with a reduced number of elements [6]. With 64 elements distributed in the SAA with a diameter of 20 mm, the transducer operates at a central frequency of 1.5 MHz. The active material of the 1–3 piezocomposite was the Pz27 piezoceramic (Ferroperm Piezoceramics A/S, Kvistgard, Denmark) and the passive material was the epoxy resin Araldit D (Ciba Geigy). The transducer has a quarter-wavelength matching layer and a soft backing layer. The maximum cross-talk measured in the frequency domain, between two adjacent elements, was −40 dB. A copper/Kevlar flex circuit bonded with non-conductive epoxy on the 1–3 piezocomposite substrate was used as electrodes of the elements of a SAA. With this technique, transducers with arbitrary element geometries could be obtained from 1–3 piezocomposite substrate by bonding a copper/Kevlar flex circuit with a non-conductive epoxy, with no need for manual soldering nor for using conductive glue for fixing the signal wires on the electrodes. As a drawback, the non-conductive epoxy layer adds spurious capacitances [6]. The thicker is the epoxy layer, the lower is the electromechanical coupling of the elements. Thus, the stress applied to the flex circuit in its bonding process is a critical task, as it determines the thickness of the epoxy layer and, therefore, the spurious capacitance.

Another approach design for reducing the number of elements in a 2-D array transducer while maintaining the wide aperture to improve the lateral resolution is the Fermat spiral [7,18]. The Fermat spiral distributes the elements in a spiral arrangement. This distribution reduces the amplitudes of the grating lobes because of the lower periodicity of the elements, even when the inter-element distances are greater than λ/2. However, its fabrication is more complex than that of a 2-D square array, because the positions of the elements in a Fermat spiral are a function of a divergence angle, that defines the angular distance between two consecutive elements [18]. The amplitude of the grating lobes depends on the divergence angle. In [18] 2-D arrays with Fermat spiral distribution, with 128 and 256 elements in apertures with diameters of 40 λ, 50 λ, and 60 λ, were simulated. In [7] a 2-D array transducer with 64 elements disposed into a Fermat spiral was manufactured by an additive process, using a 3-D printer and a thermoplastic polyurethane (TPU). The aperture was designed to have a diameter of 48 λ. The elements are squares with sides of 1.5 mm, made of 1–3 piezocomposite from Smart Material (851, dice and fill 65%). The central frequency of operation was 1.6 MHz.

To reduce the information gaps in the design and construction of transducers for 3-D underwater applications, this work presents the development of a 2-D array ultrasonic transducer for coupling in water, describing the preparation of the piezoceramics, the design and application of the matching layer and the backing layer, and the overall construction process. Every element of the array will emit and receive signals individually. This feature provides great flexibility for the implementation of many techniques of 3-D imaging.

## 2. Development of the Transducer

The construction of the 2-D array ultrasonic transducer involved the design of the two-dimensional matrix (made of 1–3 piezocomposite), the design of the matching layer, and the backing layer. Once constructed, the transducer was characterized to find the electrical impedances, central frequency, and bandwidth.

**Figure 1:**
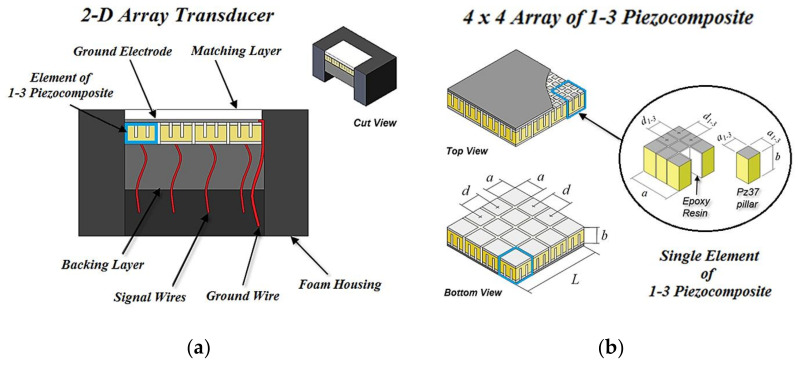
(**a**) Overview of the 2-D array ultrasonic transducer prototype; (**b**) Dimensions of the array made of 1–3 piezocomposite: *a* = 5 mm, *a_1–3_* = 1.53 mm, *d* = 5.2 mm, *d_1–3_* = 1.73 mm, *b* = 2.8 mm, *L* = 20.6 mm (a single element is outlined in blue).

An overview of the parts of the 2-D array ultrasonic transducer developed in this paper is shown in Figure 1. It consists of a 4 × 4 matrix of 1–3 piezocomposite elements, a matching layer, a backing layer, a foam housing, and wires for electrical interconnections.

The matrix of active elements, made of 1–3 piezocomposite, emits and receives the signals through the piezoelectric effect. A piezoelectric material converts electrical energy into mechanical energy (acoustical energy, in this case) and vice versa [19].

The matching layer has an intermediary acoustic impedance between the active elements and the load (water) to reduce reflections between them [19]. It increases the emission and reception energies of the signals [15,19,20,21], increasing the signal-to-noise ratio. Besides, the bandwidth also increases [1,15,20].

The backing layer can be designed to absorb the waves that propagate backward, such that the bandwidth increases. It can also be designed to reflect these waves forward by increasing the energy transmitted at the cost of reducing bandwidth [15]. The foam housing avoids environmental disturbances and supports the signals parallel connector.

### 2.1. Two-Dimensional Matrix of 1–3 Piezocomposite

The square geometry was chosen to simplify the fabrication of the 2-D array of 1–3 piezocomposite elements, due to the periodicity of the piezoelectric ceramic pillars. Even though the array has only sixteen elements arranged in a 4 × 4 grid, reducing the complexity of the electronics, this is enough to focus and deflect acoustic beams in a 3-D space, allowing one to capture 3-D images.

As the frequency decreases, the acoustic attenuation decreases, increasing the detection range [22] and the VOI. In addition to improving the range, lower operating frequencies make it possible to reduce the sampling frequency. This leads to a reduction in the computational load, because for a given time interval, the lower the sampling frequency, the less the samples and data to store and process. Perhaps this explains the relatively low operating frequencies of 3-D sonar compared to other areas of application of 3-D ultrasound imaging, such as NDE and medical. The most common operating frequencies found in the literature for 3-D sonars are 100 kHz in [5], 300 kHz in [3,4,14,17], 600 kHz in [2], and 1 MHz in [1]. Based on the literature, the central frequency chosen for the 2-D array was 500 kHz. As the transducer operates in the water, the pitch is equal to 1.7 λ, that is, the transducer is undersampled. The pitch is within the range of other planar arrays, as λ in [2], and 2 λ in [1,2,5]. For a regular array, the pitch (*d*) is given by the distance between two contiguous elements (i.e., the length of one element (*a*) added to the length of one gap), as shown in Figure 1. Considering the pitch (*d* = 5.2 mm) and the cutting thickness (kerfs = 0.2 mm), the elements have 5 mm sides.

To avoid artifacts in the images of undersampled 2-D arrays, the steering angles of the beams should be limited, both in azimuthal (θ) and elevation (φ) angles, restraining the field of view within limits, given as (in radians) [2]:

(1) $$\theta_{max}=\varphi_{max}=\pm \sin^{-1}\left(\frac{\lambda_{min}}{2d}\right)\;,$$

where λ*_min_* is the minimum wavelength that is contained in the bandwidth of the pulse.

According to Equation (1), for a given operational frequency, the higher the pitch, the smaller the field of view, as the direction of the beam boundaries is inversely proportional to the pitch. Taking into account that the sides of the elements are approximately equal to the pitch and that the theoretical central frequency of the designed 2-D matrix is approximately 500 kHz, the field of view in the horizontal and vertical directions is 33° × 33°. With the SCF technique, the field of view has been improved to reach 40° × 40°, which is compatible with the results presented in other works, such as 35° × 35° @ 1 MHz in [1], 26° × 26° @ 1.2 MHz and 52° × 52° @ 600 kHz in [2], 20° × 20° @ 2 MHz in [16], 30° × 30° @ 100 kHz in [5] and 50° × 50° @ 300 kHz in [14].

If the aperture geometry is symmetrical, that is, M = N, where M and N are the numbers of elements in width and height, the angular resolutions of azimuth and elevation are the same. The angular resolution (in radians) is determined as the width of the main lobe, according to [3,4]:

(2) $$\delta_{\theta }=\delta_{\varphi }=\frac{\lambda_{o}}{L}\;,$$

where *L* is the width of the aperture (*L* = *d*M = *d*N) and λ_o_ is the wavelength of the pulse at the central frequency of the transducer. While the angular resolution is constant, the lateral resolution is dependent on the distance, as [3,4]:

(3) $$\delta_{lateral}=r\delta_{\theta }=r\delta_{\varphi }\;,$$

where *r* is the imaging distance. This information is useful to estimate the minimum lateral distance needed to distinguish two contiguous objects at a given distance *r*. For example, a 2-D array with 20 mm sides, operating at 500 kHz, can solve two reflectors spaced 0.15 m laterally at a distance of 1 m. However, if the sonar was placed at 2 m, the objects cannot be resolved, because at this distance the lateral resolution is 0.3 m.

The range resolution (minimum distance to resolve two equal scatterers at the same direction) was calculated as [3,4]:

(4) $$\delta_{range}=\frac{c}{2\Delta f}\;,$$

where c is the propagation velocity in the water and Δ*f* is the bandwidth of the transducer.

**Figure 2:**
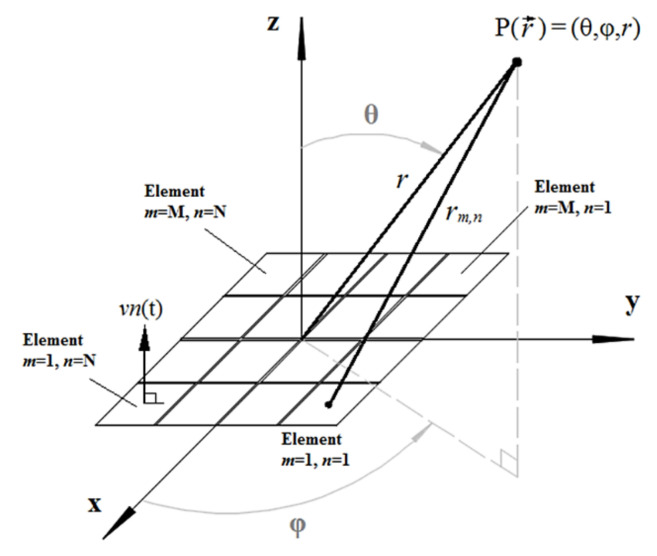
Geometry and coordinate system for impulse response calculation of the 2-D array.

The acoustic field was simulated to assess the 3-D beam deflection, and it was performed using the software Matlab^®^ (The MathWorks, Natick, MA, USA). The simulated fields are calculated by applying a particular analytical solution of the Rayleigh integral, which evaluates the velocity potential impulse response of rectangular transducers modeled as a rigid piston, surrounded by an infinite rigid baffle [23]. This model evaluates the acoustic field directly in the time domain with arbitrary and realistic excitations (wideband transient pulse, continuous wave, tone burst, and others). Furthermore, it also takes into account the geometry of the elements and the distance between them (kerfs). All of these features generate more realistic simulations. The transient pressure generated by the M × N array (consisting of single piston-like transducers) at point P ($\vec{r}$) of a homogeneous and lossless medium, as shown in Figure 2, can be calculated as [8,23]:

(5) $$p\left(\vec{r},t\right)=\rho \frac{\partial vn\left(t\right)}{\partial t}*h_{a}\left(\vec{r},t\right)\;,$$

where *ρ* is the density of the medium, *vn*(t) is the normal component of the piston vibration velocity (excitation function), *h_a_* is the contribution of the impulse response of all elements of the array, and * is the convolution operation.

The impulse response of the 2-D array, *h_a_*, is calculated by adding the individual impulse responses of all elements, *h_mn_*, as [8]:

(6) $$h_{a}\left(\vec{r},t\right)=\overset{M}{\underset{m=1}{\sum }}\overset{N}{\underset{n=1}{\sum }}A_{mn}h_{mn}\left(\vec{r},t-{\Delta t}_{mn}\right)\;,$$

where A*_mn_* is an apodization constant and Δt*_mn_* is the delay.

The delays Δt*_mn_* are included in all impulse responses to ensure that pulses reach a focal point at the same time. If the point P ($\vec{r}$) is the focal point (Figure 2), the delays will be calculated as [8]:

(7) $${\Delta t}_{mn}=\frac{r-r_{mn}}{c}\;,$$

where *r* is the distance from the center of the array to the focus point, *r_mn_* is the distance from the center of the element (*m*, *n*) to the focus point and *c* is the acoustic propagation velocity in the medium.

The pulse-echo response of an element, or a pair of transmitter/receiver elements of the array, from a pointwise reflector in P ($\vec{r}$), can be computed by assuming the output voltage to be [8]:

(8) $$V_{out}\left(\vec{r},t\right)=\rho \frac{\partial vn\left(t\right)}{\partial t}*h^{T}\left(\vec{r},t\right)*h^{R}\left(\vec{r},t\right)\;,$$

where *h^T^* and *h^R^* are the impulse responses of the transmitter and receiver elements.

Simulations of the beam deflections were made with a narrowband pulse, with 20% of bandwidth, and a wideband pulse, with 50% of bandwidth. The excitation velocity function at the transducer aperture (piston vibration velocity) was given by [23]:

(9) $$vn\left(t\right)=vn_{o}t^{3}e^{-kf_{o}t}\cos\left(2\pi f_{o}t\right)\;,$$

where *vn_o_* is the amplitude, t is the time, *f_o_* is the central frequency of the transducer, and k = 1.437 for 20% of bandwidth, and k = 3.833 for 50% of bandwidth. The parameters used for the acoustical simulations are shown in Table 1.

**Figure 3:**
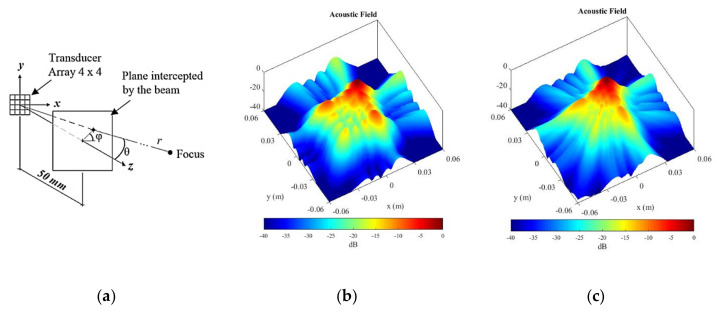
(**a**) The acoustic field of the 2-D array was simulated at a parallel plane 50 mm apart from the transducer face (far-field), focused at θ = 15°, φ = 45° and *r* = 1 m, (**b**) with narrowband and (**c**) wideband excitations, respectively.

Using spherical coordinates as shown in Figure 3a, the beam was deflected to 15° relative to the z axis and rotated 45° around the same axis. The simulations indicate that grating lobes grow and move away from the main lobe when the excitation is narrowband (Figure 3b). On the other hand, the wideband excitation seems more suitable for the array geometry designed, showing smaller grating lobes (Figure 3c).

The aspect ratio criterion to make the piezoelectric elements operate preferably in thickness vibration mode rather than lateral coupling modes is given by *a*/*b* < 0.6 (pillar or rod aspect) or *a*/*b* > 10 (disc or plate aspect), where *a* and *b* are the lateral dimension and the thickness of the element, respectively [15]. For the designed geometries of the 2-D array transducer (Figure 1), the aspect ratio of each element is 1.78, which exceeds the ideal condition.

Alternatively, elements made of 1–3 piezocomposite can be used instead of pure piezoceramics to increases the vibration of the elements in thickness mode, decreasing the lateral vibrations modes [15]. Furthermore, the acoustic impedance of piezocomposite 1–3 is less than that of pure piezoceramics, improving the acoustic matching between the transducer and water [24,25].

Each 1–3 type piezocomposite element consists of a group of piezoceramic pillars (active material) whose interspaces are filled with a passive material, generally an epoxy resin. The pillar faces of each element can be covered with conductive resin or metallic sheet, making the electrodes. The index “1” in “1–3” means that the piezoceramic phase is continuous in only one direction (z-axis), and the index “3” means that the epoxy resin phase is continuous in three directions (x-, y-, and z-axes).

The design of the matrix of 1–3 piezocomposite started from a pure Pz37 piezoceramic of 500 kHz, which is a material with a very low acoustic impedance of 18 Mrayl (Ferroperm Piezoceramics A/S, Kvistgard, Denmark). Using a 0.15-mm-thick saw blade (Buehler, Lake Bluff, IL, USA), each element was divided into nine pillars with sides equal to 1.53 mm, resulting in square-faced elements with sides of 5 mm (Figure 1). The inter-pillars spaces were filled with low viscosity epoxy resin, with an acoustic impedance of 3 Mrayl (Araldite^®^ GY 279 and Aradur^®^ 951 hardener). The aspect ratio of each pillar is *a_1–3_*/*b* = 0.55, and the volume fraction of piezoelectric ceramic in each 1–3 piezocomposite element, given by *a_1–3_*^2^ / *d_1–3_*^2^ [25], is 78%.

**Figure 4:**
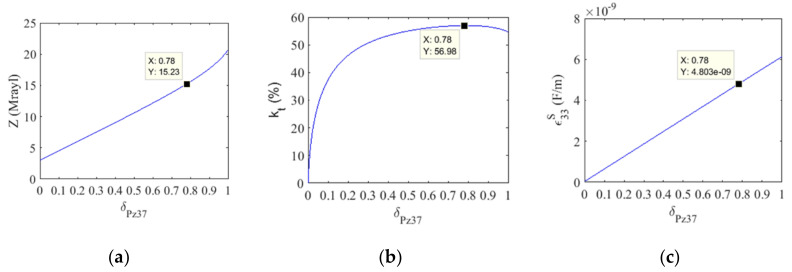
1–3 piezocomposite material parameters versus Pz37 volume fraction: (**a**) acoustic impedance, (**b**) electromechanical coupling, (**c**) dielectric permittivity. The points on the curves indicate the property values for the 78% volume fraction.

The properties of the 1–3 piezocomposite constructed in this work (acoustic impedance—*Z*, electromechanical coupling—k_t_, dielectric permittivity—$\varepsilon_{33}^{S}$) were obtained using the model described in [24], as shown in Figure 4. The use of 1–3 piezocomposite instead of pure ceramic reduces the acoustic impedance from 18 Mrayl to about 15 Mrayl when the volumetric fraction of Pz37 is δ_Pz37_ = 78% (Figure 4a). This leads to an improvement in the emission and reception of signals due to a better acoustic matching of the elements with the water. Electromechanical coupling in the thickness mode will also be increased (Figure 4b). However, the decrease in dielectric permittivity (Figure 4c) increases the reactive capacitance. This reduces the electrical matching such that less electrical energy will be transmitted to the transducer. To overcome this limitation, the reactive capacitance will be canceled close to the resonance frequency using a series inductor to match the electrical impedance.

### 2.2. Acoustic Matching Layer and Backing Layer

To improve the bandwidth, as well as the emission and reception of signals, a layer of a composite made of alumina powder embedded in epoxy resin (the same type of epoxy resin used in the 1–3 piezocomposite) may be added to the transducer face. The acoustic impedance of the matching layer was calculated by the geometric mean of the acoustic impedances of water and the 1–3 piezocomposite [19,20,21], resulting in 4.8 Mrayl.

**Figure 5:**
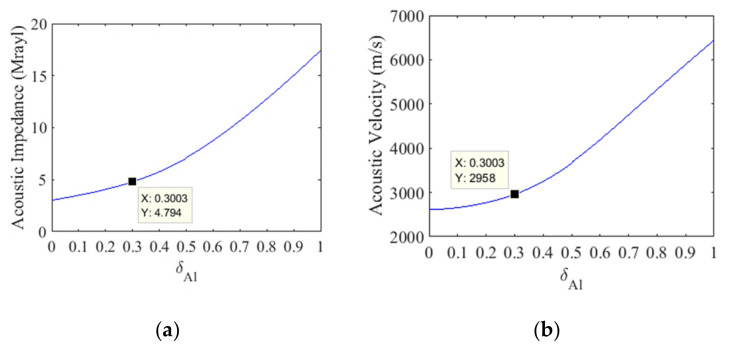
(**a**) Theoretical curves of the acoustic impedance and (**b**) the propagation velocity were used to determine the alumina volume fraction and the matching layer thickness.

The model by Sayers and Tait [26] is used to determine the theoretical curves of the acoustic impedance and the propagation velocity of the composite mixture of particles of alumina and epoxy resin, as shown in Figure 5. From these curves, the *δ_Al_* volume fraction of alumina particles to be mixed in the epoxy resin is found, which is the composite mass of the acoustic matching layer of the transducer. Initially, the volumetric fraction of alumina powder (*δ_Al_* = 30%) was found, corresponding to the acoustic impedance of the matching layer, according to Figure 5a. Next, the acoustic propagation velocity of the matching layer is determined directly from the alumina volume fraction, using the curve shown in Figure 5b. Then, the quarter-wavelength-thick matching layer is calculated at the central frequency of the transducer (*f_o_*) considering the propagation velocity of 3003 m/s, resulting in close to 1.5 mm.

The choice of the backing layer material must take into account the trade-off between the bandwidth and sensitivity of signals [15,21]. If broad bandwidth signals are desired, a backing layer with acoustic impedance close to that of the piezoelectric element and high acoustic attenuation should be used to reduce the pulse size [15,21,26]. One of the most common materials used for this purpose consists of tungsten powder embedded in epoxy resin [26]. However, a high attenuation backing layer dissipates the acoustic energy that could be transmitted to the medium, reducing the capability of the sonar to detect distant objects. Therefore, a backing layer with an acoustic impedance lower than that of the piezoelectric element is more suitable for underwater sonars, increasing the energy to be transmitted forward [1].

An air backing layer can be a perfect reflector of the backward waves generated by the transducer, improving the sensitivity of the transducer. This happens because the acoustic impedance of the air is much lower than that of piezoelectric ceramics. However, in deep waters, the air backing layer can collapse the transducer due to the high hydrostatic pressure. Also, a perfect seal of the air backing of a transducer designed to operate in a water environment can be a critical task.

Instead of using air in the backing, low acoustic impedance materials can also be used to obtain a compromise between emission and mechanical resistance. A backing layer of epoxy resin was tested in this paper, because it has a low acoustic impedance, and provides mechanical resistance. A sample was characterized by the ultrasonic spectroscopy technique described in [27,28].

The knowledge of the pressure transmission and reflection coefficients is useful to choose the type of material of the backing layer. If the characteristic acoustical impedance of the piezoelectric element (Z*_piezo_*) and the backing layer (Z*_backing_*) are known, the acoustic pressure transmitted from the piezoelectric element to the backing layer, and the pressure reflected back at the backing layer/element interface, can be evaluated. This is calculated using the pressure transmission and reflection coefficients, that for a normal incidence of longitudinal waves are given, respectively, by [20,21]:

(10) $$T_{p}=\frac{2Z_{backing}}{Z_{piezo}+Z_{backing}}\;,$$

(11) $$R_{p}=\frac{Z_{backing}-Z_{piezo}}{Z_{piezo}+Z_{backing}}\;,$$

On the other hand, to know the amount of energy transmitted from the piezoelectric element to the backing layer and reflected back to the element, the acoustic intensity transmission, and reflection coefficients are given as [20,21]:

(12) $$T_{i}=\frac{4Z_{piezo}Z_{backing}}{{\left(Z_{piezo}+Z_{backing}\right)}^{2}}\;,$$

(13) $$R_{i}=1-T_{i}\;,$$

Two types of backing layer materials—air and epoxy resin—were used to build transducers T1 and T2, respectively. The main parameters of the backing layers used in the prototypes are presented in Table 2. For the transducer with air backing (T1), all the energy that propagates backward reflects on the air-ceramic interface (*R_i_* = 1), improving the emission. In the case of the transducer with an epoxy resin backing (T2), only 50% of the energy of the waves that propagate backward contribute to the emission (*R_i_* = 0.51).

## 3. Construction of the Transducer

**Figure 6:**
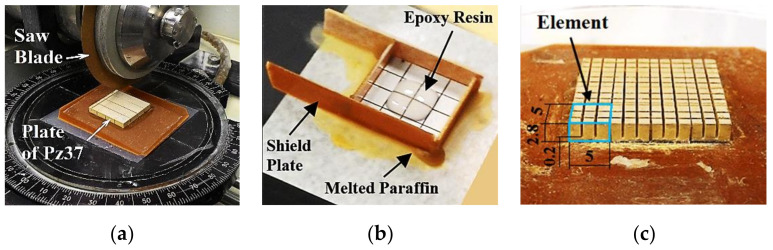
(**a**) The 4 × 4 array was made dicing the Pz37 plate in orthogonal directions at steps of 5.2 mm (**b**) and the kerfs were filled with epoxy resin. (**c**) 1–3 piezocomposite elements with nine pillars were made repeating the dice-and-fill technique with kerfs at steps of 1.73 mm. A single element is outlined in blue.

The construction began with the fabrication of the 4 × 4 matrix of pure piezoceramic. The plate of Pz37 piezoceramic of 500 kHz was diced in orthogonal directions, with a semiautomatic ISOMET 4000 precision cutting machine (Buehler, Lake Bluff, IL, USA) (Figure 6a). The elements were separated by dicing the plate to produce a pitch of 5.2 mm (element side of 5 mm plus cutting thickness of 0.2 mm). This cutting thickness was achieved by using a 150-μm-thick saw blade, because vibrations end up increasing the cutting thickness. The rotation of the blade was set at a high speed (5000 rpm) and the forward cutting rate was set at a very low speed (2.5 mm per minute) in order to minimize the cutting errors, thus ensuring reproducibility. The rotation was only not higher to avoid cracks in the ceramic and in the saw blade. Besides, the cutting process was carried out with water lubrication. The inter-element spaces were filled with epoxy resin Araldite^®^ GY 279 and hardener Aradur^®^ 951 from Huntsman International LLC (Figure 6b). To avoid resin leakage during the filling process, a thin phenolic plate shield held with wax and melted paraffin was attached to the sides of the array. This process of cutting ceramics and filling gaps (kerfs) with epoxy resin is known as the dice-and-fill technique [6,8,11].

Next, again using the dice-and-fill technique, the pillars of 1–3 piezocomposite elements were made with kerfs at steps of 1.73 mm, but at that moment an overlayer of 0.2 mm thickness was left every two cuts to maintain the electrodes of the elements and the position of the pillars. This allows the maintenance of the original electrodes in those regions, simplifying the process. The cutting process resulted in nine pillars for each element of the matrix (Figure 6c). All electrodes of the pillars were joined with CW2400 conductive resin (Chemtronics, Kennesaw, GA, USA) to make the ground electrode, and the square electrodes on the opposite face of the array, each one joining nine pillars, were maintained to solder the signal wires.

The matching layer was made of a composite of alumina powder embedded into epoxy resin, with 30% of alumina volume to achieve the calculated acoustic impedance of 4.8 Mrayl. The same type of low viscosity epoxy resin used to fill the array kerfs was used to provide a bubbleless homogeneous mixture. The bubbles must be avoided because they change the acoustic impedance of the matching layer and introduce reflections and scattering, which reduce the acoustic energy transmission and reception.

**Figure 7:**
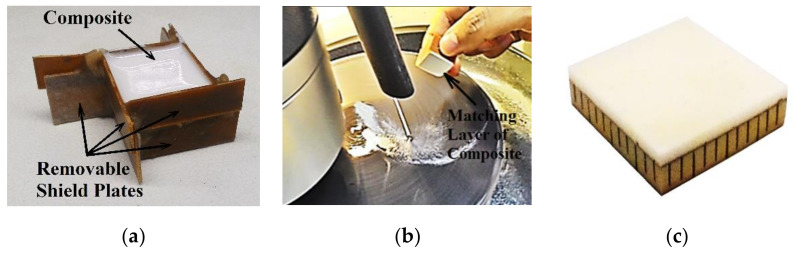
(**a**) The alumina composite was deposited over the ground electrode. (**b**) After it was cured, the composite was manually grinded until reaching the λ/4 thickness. (**c**) Picture showing the accomplished acoustic matching of the 1–3 piezocomposite array.

The composite mixture was deposited over the ground electrode of the transducer array (Figure 7a). After the resin was cured, the composite was manually ground on a TEGRAMIN-25 rotary grinding machine (STRUERS, Struers A/S, Ballerup, Denmark) (Figure 7b), until the matching layer thickness equal to 1.5 mm was obtained (Figure 7c).

**Figure 8:**
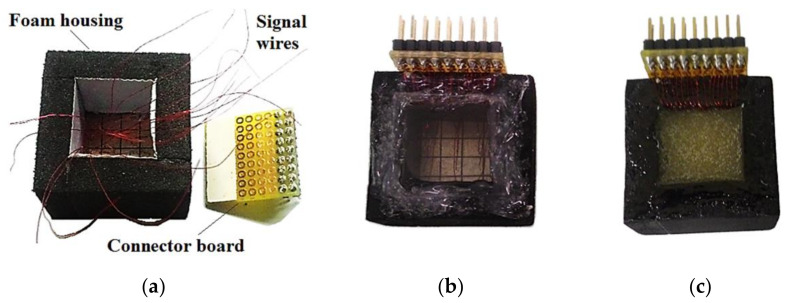
(**a**) Enameled 150-μm-diameter wires were soldered from the signal electrodes to the connector board. (**b**) The transducer T1 was obtained by closing the backing with an acrylic plate to maintain an air layer and (**c**) transducer T2 was obtained filling the backing with epoxy resin.

All the electrical interconnections of elements were made with enameled wires of diameter equal to 0.15 mm. One wire end was soldered onto the electrode of the element, and the other was soldered onto the signal connector of the board (Figure 8a). The housing was made of foam tape, which was cut into four strips and bonded with LOCTITE^®^ instant adhesive.

The backing layer of air was obtained by closing the back of the transducer with a transparent acrylic plate bonded with silicon glue, generating the transducer T1 (Figure 8b). The transducer T2 was made filling the backing with Araldite^®^ AW 106 epoxy resin and HV 953U hardener from Huntsman International LLC, resulting in a 20-mm-thick backing layer (Figure 8c).

**Figure 9:**
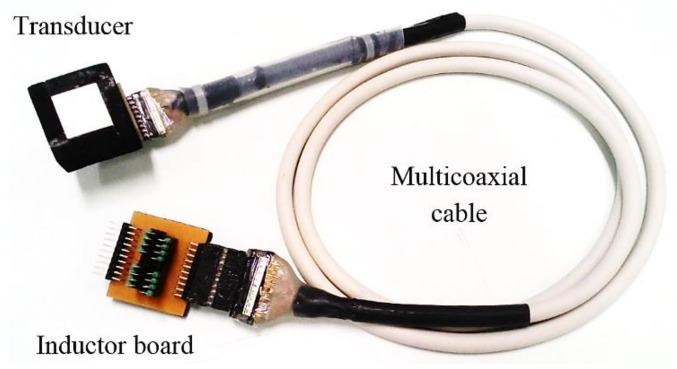
Complete transducer, with multicoaxial cable and board of matching inductors.

A board with sixteen axials 330 μH series inductors was connected to the multicoaxial cable to matching the electrical impedances. The accomplished 2D phased array prototype transducer is shown in (Figure 9).

## 4. Characterization

**Figure 10:**
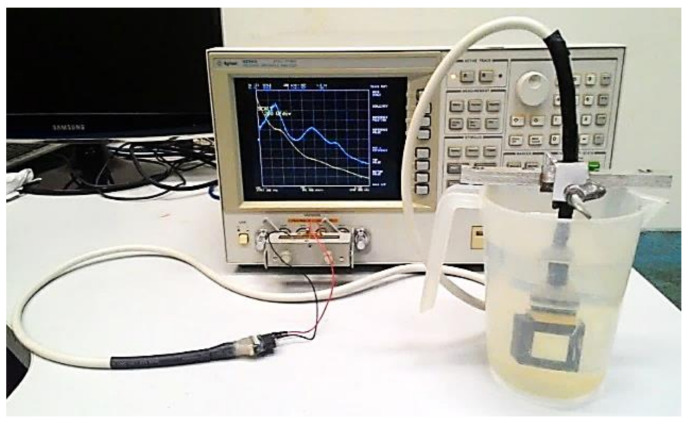
Electrical impedance measurements, |Z| (Ω) in yellow and phase (^o^) in blue was carried out with an impedance analyzer, with the transducer immersed in water, to taking into account the acoustic load of the medium of operation.

The electrical impedances were measured individually, element by element, with a 4294 A impedance analyzer (Agilent Technologies, Inc., Santa Clara, CA, USA). Considering that the transducers are for underwater applications and that the electrical impedances measured in the air are very different from those measured in the water, it´s important to take into account the acoustic load of the water in the measurements (Figure 10).

For example, the average magnitudes and phases of electrical impedance measured in air of the transducers T1 and T2 at the resonance frequency were 4058 Ω ∠ −31° @ 545 kHz and 5128 Ω ∠ −44° @ 548 kHz, respectively, whereas the measurements carried out into water were 1261 Ω ∠ −80° @ 536 kHz and 1230 Ω ∠ −82° @ 540 kHz, respectively. The electrical impedances measured of the elements are much higher than the input impedance of a typical instrument, such as the pulser/receiver unit, typically 50 Ω.

**Figure 11:**
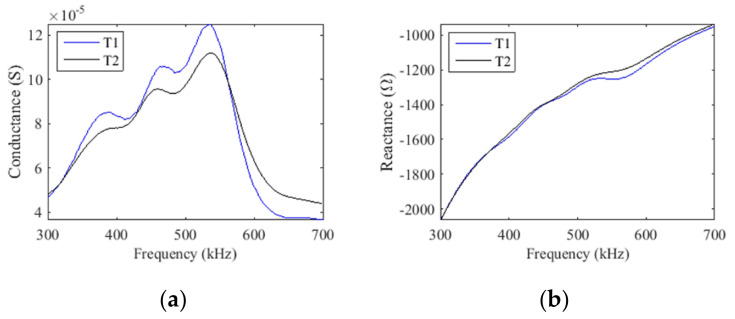
The electrical impedance measurements allow the identification of the resonance frequency at (**a**) the maximum conductance and (**b**) the reactance was used to calculate the matching series inductor (a measurement of the element 2,3).

Therefore, for maximum power transmission efficiency, it’s necessary to match the electrical impedance of the transducer with the source/input [15,19,20,21]. A method to do this is adding a series inductor to null the capacitive reactance [15,19,20,21]. The series inductor was calculated as L = X/2π*f*_o_, where X is the negative reactance measured at the resonance frequency, *f_o_*, which is found where the conductance is maximum [29] (Figure 11a). The series inductors of transducer T1 and T2 evaluated with the average measured reactance (Figure 11b), were 368.8 µH and 359 μH, respectively.

**Figure 12:**
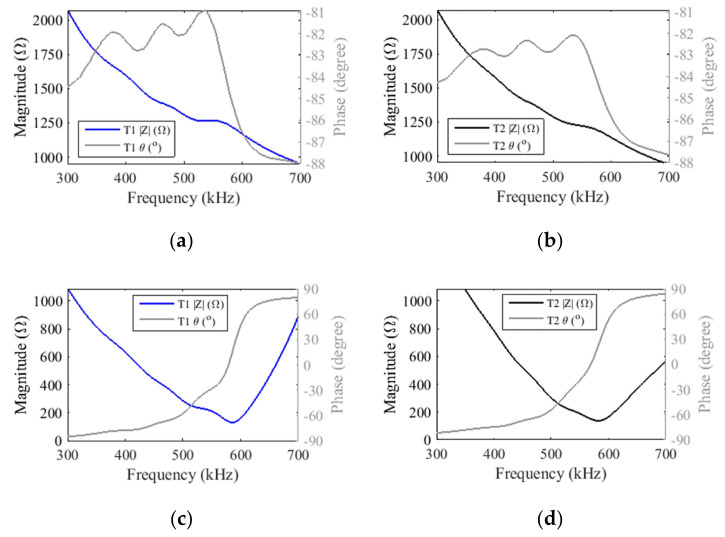
Electrical impedance magnitudes and phases of element (2,3) measured for mismatched transducers: (**a**) T1 and (**b**) T2: and for transducers with a series inductor: (**c**) T1 and (**d**) T2. Transducer T1 has air backing and transducer T2 has epoxy resin backing.

Because the values of the evaluated series inductors are similar, good results were obtained using standard axial inductors of 330 ± 10% μH in both transducers T1 and T2. With the series inductors, the electrical impedance magnitudes of both transducers T1 and T2 dropped down about ten times, achieving 135 Ω @ 588 kHz and 142 Ω @ 581 kHz, respectively, and the phase measured close to the resonance was canceled, as expected (Figure 12).

The sensitivity of the echoes, the bandwidth, and the central frequency of transducers T1 and T2 were characterized one at a time using pulse-echo measurements. Each element was driven by a Panametrics NDT 5077PR pulser/receiver unit (Olympus NDT INC., Waltham, MA, USA). The pulser/receiver unit settings were 100 Volts negative rectangular pulse with a length of T/2 = 1 µs (appropriate for a 500 kHz transducer), 0 dB reception gain, and 10 MHz low pass filter.

**Figure 13:**
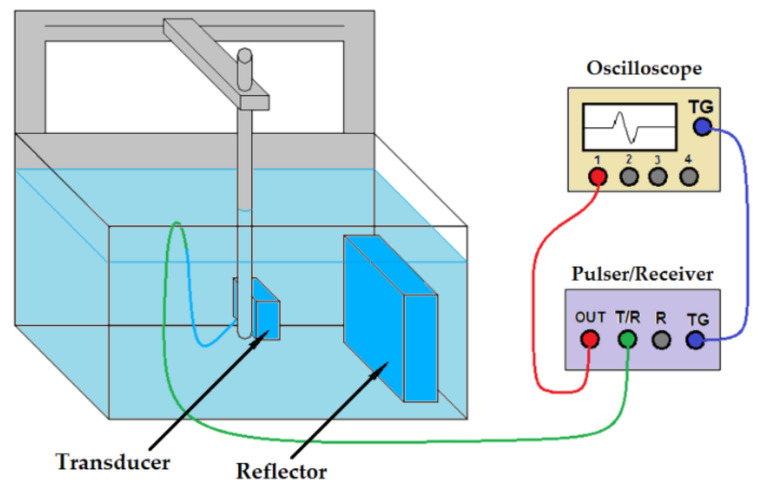
Schematic diagram of the pulse-echo measurement.

Each element of each transducer emitted a pulse that reached a plane brass reflector at 50 mm from the transducer face, as shown in Figure 13. Each echo generated was acquired by the pulser-receiver unit through its connector T/R (operating in pulse-echo mode). Each signal was then filtered by the 10 MHz low-pass filter and transmitted through the RF out connector to channel 1 of the oscilloscope Agilent Infiniium MSO8104A (Agilent Technologies, Inc., Santa Clara, CA, USA). Each signal was sampled at 50 MHz averaging 64 waveforms to avoid random noises. The pulser-receiver unit and the oscilloscope were synchronized using a cable to interconnect the trigger (TG) terminals.

**Figure 14:**
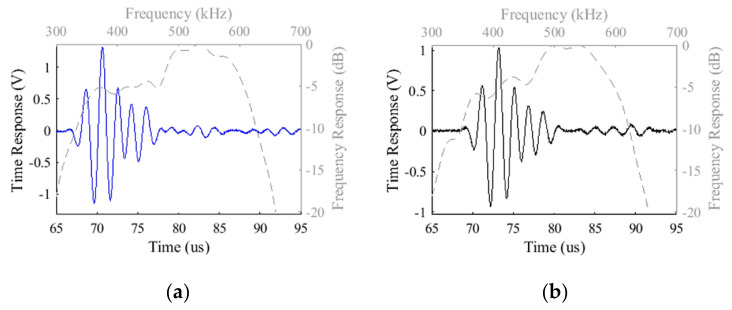
The echoes responses from a central element (*m* = 2, *n* = 3) of (**a**) the transducer T1 (air backing) and (**b**) T2 (epoxy resin backing) excited by a single rectangular negative pulse show that the air backing improves the echo amplitude (the frequency responses of all the elements were normalized to calculate the bandwidth at −6 dB from the maximum).

The peak-to-peak voltage of each element (*m*,*n*) was obtained from the waveform of the echoes (Figure 14), which were loaded in Matlab^®^. The central frequency of each element was obtained with the normalized magnitude of the FFT of the echoes plotted in dB, taking the lowest and the higher frequencies, *f*_1_ and *f*_2_, respectively, in the range where the amplitudes are equals to or greater than −6 dB. The central frequency is *f*_o_ = (*f*_1_ + *f*_2_)/2, and the bandwidth in percentage is Δ*f* = 100 x (*f*_2_ − *f*_1_)/*f*_o_.

The main characteristics of transducers T1 and T2 are summarized in Table 3.

The echoes generated by the transducer T1 (Figure 14a), which has air backing, were stronger than the echoes of the transducer T2, which has epoxy resin backing (Figure 14b). This was expected, because the acoustic transmission coefficient of 1–3 piezocomposite for the air backing, is insignificant both in amplitude and energy, reflecting all the backing waves forward. On the other hand, the epoxy resin backing can reflect 72% and 51% of the backing waves’ amplitudes and energy, respectively, generated by the 1–3 piezocomposite elements (Table 2).

Since the acoustic impedance of the air (400 rayls is considered in this work) and epoxy resin AW 106 (characterized as 2.5 Mrayls) are much lower than that of 1–3 piezocomposite (15.2 Mrayls, obtained by simulation), and a matching layer was added on the front of the transducer for matching the acoustical impedances of the 1–3 transducer and the medium (water) and improving the bandwidth, the average bandwidth of the transducers T1 and T2 were very similar, with 54% and 50% @ −6 dB, respectively.

Even though the thickness of the ceramic was not changed, the central frequency reduction from 500 kHz to 480 kHz can be attributed to a conversion of the pure piezoceramic into a 1–3 piezocomposite with different effective properties (as shown in Fig. 4). The conversion process applies the dice-and-fill technique, which ends up replacing part of the ceramic by epoxy resin.

The peak-to-peak voltages and the bandwidth of the echoes measured with transducers T1 and T2 were compared using a function of sensitivity, given by:

(14) $$\mathrm{Sensitivity}\left(j,m,n\right)=20\times \log_{10}\left(\frac{measure\left(j,m,n\right)}{overall\_mean}\right)\;,$$

(15) $$overall\_mean=\frac{1}{J\times M\times N}\overset{J}{\underset{j=1}{\sum }}\overset{M}{\underset{m=1}{\sum }}\overset{N}{\underset{n=1}{\sum }}measure\left(j,m,n\right)\;,$$

where *j* is 1 or 2, if the measure corresponds to transducer T1 or T2, respectively, *m* and *n* are the rows and column coordinates of the positions of the elements, *measure*(*j*,*m*,*n*) is the Peak-to-Peak voltage or the bandwidth measured from the element (*m*,*n*) of the transducer *j*, and *overall_mean* is the arithmetical mean from a given type of measurement (peak-to-peak voltage or bandwidth) from all elements, including both transducers T1 and T2.

**Figure 15:**
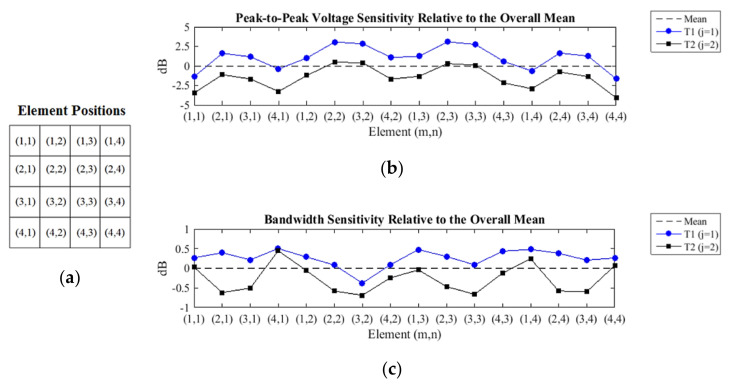
(**a**) The element positions are addressed as (m = line, n = column). (**b**) The transducer T1, j = 1, provides higher echo amplitudes and (**c**) broader bandwidth, being almost all of them above the overall mean, whereas the measurements of the transducer T2, j = 2, have shown worse results, mostly below the overall mean.

The sensitivity of the peak-to-peak voltage shows that most elements of the transducer T1 have amplitudes above the overall average, while the voltage sensitivity of the T1 transducer echoes is constantly 2.6 dB higher than that of the transducer T2 (Figure 15a). This is consistent because the transducer T2 was created from the transducer T1 only by the addition of a layer of epoxy resin with a thickness of 20 mm, without altering any other constructive characteristics.

As with the results obtained in the sensitivity of the peak-to-peak voltage, the sensitivity of the bandwidth shows that the elements of the transducer T1 have above average bandwidth, and always above those obtained with the elements of the transducer T2 (Figure 15b). The variation of bandwidth sensitivity between elements of transducers T1 and T2, in the same position, was not homogeneous, contrary to what happened with the sensitivity of the peak-to-peak voltage.

**Figure 16:**
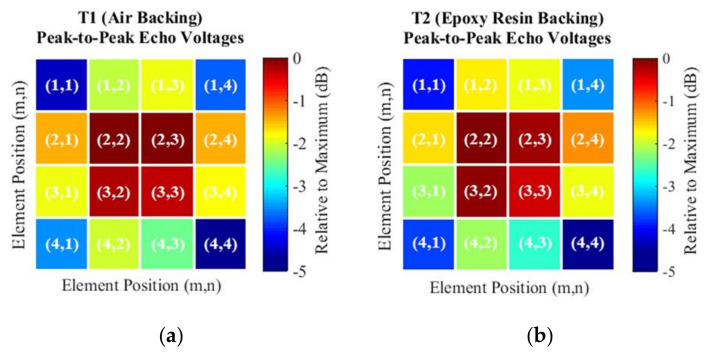
The echo responses of (**a**) the transducer T1 and (**b**) transducer T2 present an apodization effect, where the peripheral elements that are connected to the foam housing (soft material) present amplitudes lower than those of the central elements, surrounded by piezoelectric elements (hard material).

The oscillatory behavior of the measured sensitivities presented in Figure 15 is due to the apodization effect, which attenuates the amplitudes of the echoes of the peripheral elements in relation to the central elements (Figure 16). Maybe this phenomenon occurs due to the soft housing material, so it does not restrain the lateral modes of vibration, causing a loss of energy in the emission. On the other hand, the soft housing material reduces the disturbances induced by the medium.

Reproducibility of the production of the proposed transducers can be guaranteed, as long as the same piezoelectric ceramic, the same epoxy resin, the same mixtures for backing and matching are used. These are the main factors that influence the resonance frequency. The small variations in the thickness of the cuts (made to separate the elements and to produce the pillars of the 1–3 piezocomposite) can be assumed as negligible. They should not produce a relevant change in bandwidth, resonance frequency, and electrical impedance of the transducer, as well as in the acoustic field generated by the transducer.

## 5. Tests of 3-D Imaging with the Transducer T1

Images of flat targets immersed in water were carried out with transducer T1, because it presented greater sensitivity and bandwidth than transducer T2. The delay-and-sum technique (DAS), which combines the A-scan measurements of emitted and received pulses by the elements of the array to form directive beams directly in the time domain [2,3,5], was used to make images.

The A-scan signals were acquired with the full matrix capture (FMC) technique. This technique consists of storing all possible combinations of emission and reception of signals by the array (if a 2-D array has N elements, N^2^ measurements are realized), making feasible testing many types of imaging algorithms.

The acquisition was obtained exciting each element with the pulser/receiver unit adjusted with the same parameters used in the pulse-echo characterization. The echoes were one-by-one manually measured because the NDT 5077PR pulser/receiver is single-channel, therefore, 256 measurements for each image reconstructed were required. Although, if a parallel multichannel system were used the FMC data set could be obtained with only 16 pings, because for each single element emission the backscattered echoes would be acquired by all elements at the same time, in parallel.

All data stored in the oscilloscope was transferred to a PC, where the signals were digitally filtered and the images were carried out with software implemented in Matlab^®^. The signals were digitally filtered by a band-pass Hamming windowed-sinc filter. The lower and the upper cutoff frequencies were 0.5 *f_o_* and 1.5 *f_o_*, respectively, being *f_o_* the central frequency of the transducer.

Two types of beamforming technique were realized from the FMC data set: total focusing method (TMF), which is considered the gold-standard for DAS beamformers [30], and a single-ping beamformer, which emits an omnidirectional pulse from a single transmitter and receives the backscattered signals with the all elements of the 2-D array [2,3,16].

The TFM improves the signal-to-noise ratio at cost of time processing due to the amount of data and the time of flight of the sound in the water. A single-ping imaging technique decreases the quality of images, if the same array is used, but is suitable for images in real-time. In both beamformers, the pulses emitted are considered omnidirectional, because the targets are in the far-field, at distances *r* > *L*^2^/2λ_o_, where *L* is the width of the square 2-D array, or its diameter if the transducer were circular [3,16].

**Figure 17:**
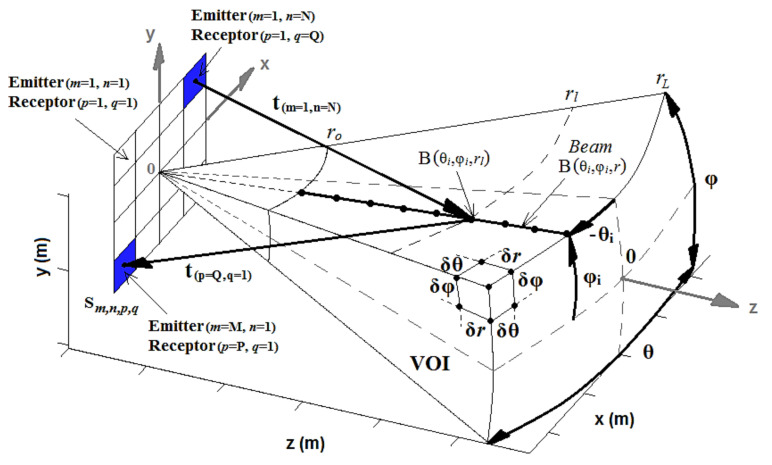
Coordinates system of the “pyramidal” volume of interest (VOI), where the 3-D images are formed.

In essence, the 3-D images approached in this paper are point cloud range images generated within a “pyramidal” volume of interest (VOI). The VOI is constituted from beams, B(θ*_i_*,φ*_i_*,*r*), which are deflected at *i* directions driven by the azimuthal (θ*_i_*) and elevation (φ*_i_*) angles (Figure 17).

Either in the TFM or single-ping beamformer, each directive beam is formed by focusing dynamically in δ*r* steps, from *r_o_* to *r_L_*. The *r_o_* distance must be large enough to avoid the oscillations (rings) generated at the beginning of the sampling, while *r_L_* must be below the total sampled window of the measured A-scans (*r_L_* < t*_sample_* c / 2). In summary, a beam B(θ*_i_*, φ*_i_*, *r*) is a vector, whose samples are the result of dynamic focusing, such as each sample is calculated as:

(16) $$B\left(\theta_{i},\varphi_{i},r_{l}\right)=\overset{M}{\underset{m=1}{\sum }}\overset{N}{\underset{n=1}{\sum }}\overset{P}{\underset{p=1}{\sum }}\overset{Q}{\underset{q=1}{\sum }}S_{m,n,p,q}\left(t+{\Delta t}_{m,n,p,q}\right)|{}_{t=0}\;,$$

where S*_m,n,p,q_* is the measured signal with the element (*p, q*) centered at (x*_p,q_*, y*_p,q_*, z*_p,q_*), from the emitted pulse with the element (*m, n*) centered at (x*_m,n_*, y*_m,n_*, z*_m,n_*), and Δt*_m,n,p,q_* is the delay of focalization, that is simply the time of flight from the pulse emitted by the element (*m, n*) up to *r_l_* and its return to the receiver element (*p, q*), that is computed as:

(17) $${\Delta t}_{m,n,p,q}=\left(t_{m,n}+t_{p,q}\right)\;,$$

(18) $$t_{m,n}=\frac{\sqrt{{\left(x_{m,n}-x_{i}\right)}^{2}+{\left(y_{m,n}-y_{i}\right)}^{2}+{\left(z_{m,n}-z_{i}\right)}^{2}}}{c}\;,$$

(19) $$t_{p,q}=\frac{\sqrt{{\left(x_{p,q}-x_{i}\right)}^{2}+{\left(y_{p,q}-y_{i}\right)}^{2}+{\left(z_{p,q}-z_{i}\right)}^{2}}}{c}\;,$$

(20) $$\left[\begin{array}{c} x_{i}\\ y_{i}\\ z_{i} \end{array}\right]=r_{l}\left[\begin{array}{c} \cos\left(\varphi_{i}\right)\sin\left(\theta_{i}\right)\\ \sin\left(\varphi_{i}\right)\\ \cos\left(\varphi_{i}\right)\cos\left(\theta_{i}\right) \end{array}\right]\;,$$

After all the beams are formed, the envelope is calculated with the Hilbert transform, normalized to the highest amplitude between all beams, and logarithm compressed. The images are realized by searching in each *i* deflected beam, the sample “*r_l_*” where the amplitude is the maximum. To avoid false detections due to low signal-to-noise ratio when the objects are not near enough to be detected, out of the field of view, or any other case that only a random noise is detected, a threshold is utilized. The range image at a given *i* direction is the linear distance, *r_l_*, determined as:

(21) $$I_{range}\left(\theta_{i},\varphi_{i},r\right)=\left\{\;\begin{array}{cc} r_{l}, & \max\left[B\left(\theta_{i},\varphi_{i},r\right)\right]\geq \mathrm{Threshold}\\ r_{L}, & \max\left[B\left(\theta_{i},\varphi_{i},r\right)\right]<\mathrm{Threshold} \end{array}\right.\;,$$

The 3-D images are depicted by converting the I*_range_* to Cartesian coordinates using Equation (20). Thus, the image can provide the position of the discretized targets surfaces, furthermore, provides the linear distance (radius) of the targets in given azimuthal and elevation angles, with the graduated color bar (red for the nearest objects and blue for the most distant).

The radius step was chosen as half of the range resolution, that is, δ*_r_* = δ*_range_*/2 = 1.5 mm, and the angle step of both δθ and δφ was 1°. Since the aperture is symmetrical, both the azimuth and elevation deflection limits to avoid the grating lobes are ±16.5° (Equation (1)), that is, the field of view is 33° × 33°. However, as the bandwidth of the transducer T1 was wide enough to allow the implementation of the sign coherence factor (SCF) filter [30,31], artifacts due to grating lobes were avoided in images within up to 40° × 40° field of view. The limit of the field of view was obtained by adjusting the sensitivity exponent of the SCF equation, which is detailed in [30,31], until finding a value that the artifacts were not present in the images. The higher the exponent, the higher the suppression of side lobes and grating lobes, and the narrower the main lobe [31]. However, the higher the exponent, the lower the detection of weak reflectors (the ones with acoustical impedance near the water, the most distant reflectors, or those whose echoes were generated by an oblique incidence). On the other hand, the smaller the exponent, the more progressively it approaches the original image until it is reached when the exponent is equal to null [31].

**Figure 18:**
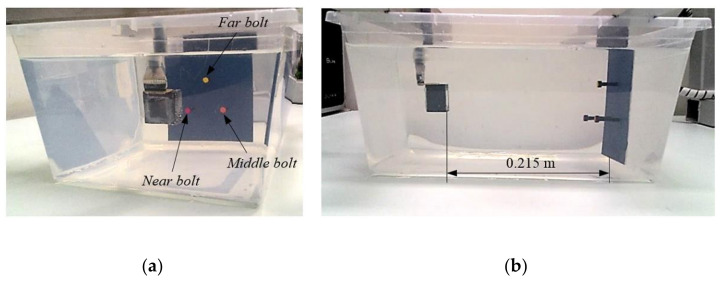
(**a**) Front view and (**b**) Side view of the three-dimensional experiment using three steel bolts at 120° between them. The height of the red, orange, and yellow bolts are 35, 30 and 25 mm, respectively, and the diameter is 8.5 mm (≈2.5 λ).

The first experiment had the purpose of assessing images of targets at different lateral, height, and depth positions between them (Figure 18).

Because the amplitudes of the echoes generated by the support plate were much higher than that of the targets, whose diameters are on the order of a wavelength (≈2.5 λ), and the threshold was defined concerning the overall maximum amplitude of the beams, it was necessary to segment the image by limiting the range to *r_L_* < 0.215 m, to avoid hiding the targets by the echoes from the support plate. The range was *r*_o_ = 0.160 m to *r_L_* = 0.210 m (*r* = 0.050 m), resulting beams with 33 points. The field of view was set to the maximum, 40° × 40°, resulting in 451 beams/direction. The SCF exponent was set to 1.2.

**Figure 19:**
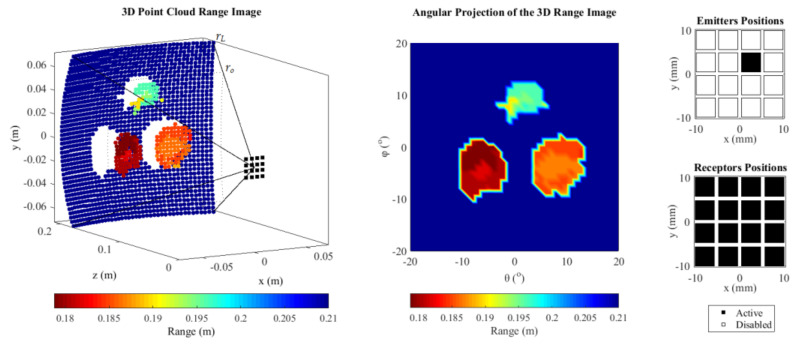
Images formed with all echoes generated by the emission of one element (m = 2, n = 3) and threshold of −20 dB.

The single-ping images were realized by evaluation of the beamforming (Equation (16)) from the FMC data set, using only the echoes generated from one element (m = 2, n = 3). Even if the circular geometry of the targets was not clearly identified, the targets were easily located in three-dimensions (Figure 19).

**Figure 20:**
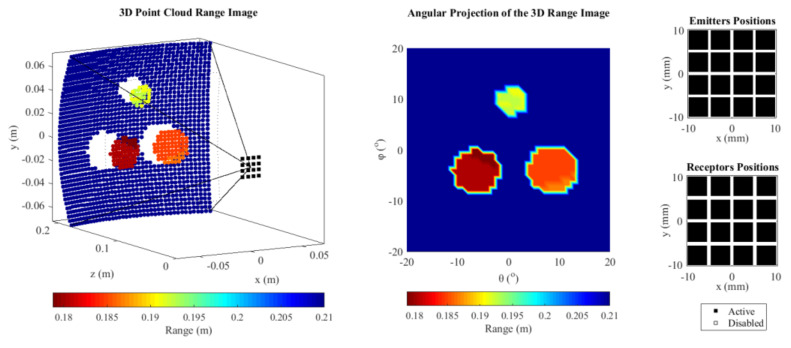
The images formed from all FMC data sets improved the geometric definition of the targets. The greater amount of signals decreased the threshold from −20 dB to −30 dB.

On the other hand, the images taken with the TFM technique showed a better definition of the targets, since all data from the FMC were used (Figure 20). The TFM increased the signal-to-noise ratio, increasing the threshold to −30 dB, instead of the −20 dB required by the single-ping beamformer. This is an advantage, because the deeper the threshold, the more targets can be visualized in the same VOI.

However, the TFM was much slower than the single-ping technique to obtain an image of the same VOI. This occurred because the beamforming given by Equation (16) is dependent on the number of emitters/receivers signals. Using a linear approach, it was expected that the time to make a TFM image could be sixteen times the single-ping image, although this has not occurred. In an average of four images with both techniques, the TFM image had eleven times the processing of single-ping.

Besides, it was verified that after all beams were formed, the time to compute the I*_range_* with the TFM and with the single-ping technique is equals, because Equation (21) does not depend on the number of emitters/receivers signals. Also, the time to calculate the I*_range_* was only 3.2% and 36% of the beamforming time with the TFM and single-ping, respectively. These results can be useful for the development of an optimized algorithm to make 3-D images since it is shown that the greatest computational load occurs in the beamforming process, therefore, it is the main critical issue to be solved. Perhaps, the beamforming time can be reduced by making only the beams deflections, without the dynamic focusing. This can be done by calculating a single delay for each emitter/receiver signal for each azimuth/elevation direction, fixing the focus at a distance much greater than the *r_L_* distance, and then adding the delayed signals coherently.

**Figure 21:**
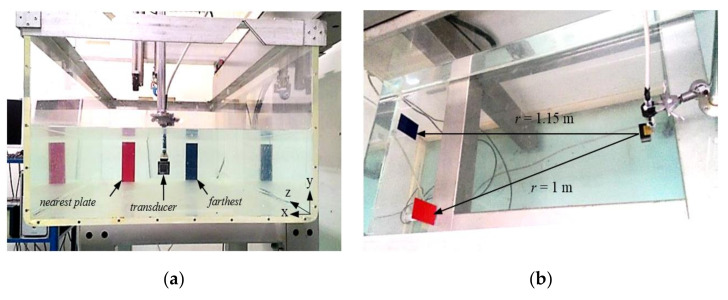
(**a**) Front view and (**b**) Top view of the experiment with faraway targets: pulse echoes from two aluminum plates of 60 mm × 160 mm immersed in a water tank of 1.4 m x 0.6 m filled up 0.2 m, were acquired with the 2-D array.

The second experiment had the purpose of assessing images of more distant targets and more interspaced in lateral position and depth (Figure 21). The experimental images were limited to 1.15 m from the transducer due to the length of the tank. All beams were formed in the range from *r*_o_ = 0.9 m to *r_L_* = 1.25 m (*r* = 0.35 m), resulting in vectors with 225 points in each direction. The field of view in the azimuth was 40°, while the elevation was limited to 10° due to the height of the objects and the water column, resulting in 451 beams. The SCF exponent was set to 1.5.

**Figure 22:**
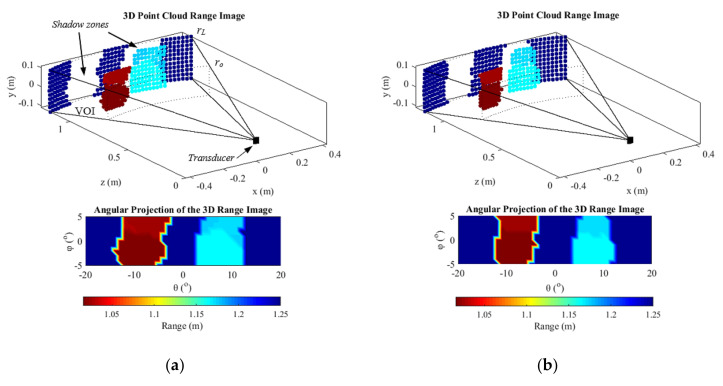
Images made from (**a**) a single ping of the element m = 2, n = 3 required a higher threshold level than that with (**b**) the TFM, −20 dB, and −30 dB, respectively. Furthermore, the objects were larger in the single ping image, decreasing the angular resolution.

The single-ping images were formed as in the first experiment, using only the echoes generated from the element (m = 2, n = 3). Although it has been possible to make images of targets more than one meter away using a single element emission (Figure 22a), its resolution was lower than that obtained with the TFM technique (Figure 22b), making the objects look wider and closer to each other. As in the first experiment, the dynamic range was improved by 10 dB with the TFM technique.

The 3-D image generated with TFM was ten times slower than with the single-beam technique. The I*_range_* calculation time was the same for both TFM and single-ping techniques, and was 0.9% and 10.4% of the beamforming time with the TFM and single-ping technique, respectively.

Both the first and second 3-D experimental images showed that the beamforming time increases when the number of emitting/receiving signals increases, but this relationship was not linear as the beamforming Equation (16), when it was computed in the software developed in Matlab^®^. Even though the TFM had sixteen times the number of emitter/receiver signals from the single-ping technique, the TFM beamforming time was eleven times and ten times the single-ping beamforming time in the near and far range images, respectively.

The first experimental images were taken 1.8 and 1.6 times faster than the second ones, with single-ping and TFM beamforming techniques, respectively (the results were obtained with an average of four). It is can be explained by the number of iterations necessary to calculate the beamforming (Equation (16)), which is directly proportional to the number of samples of the VOI. While the first VOI (Figure 19 and Figure 20) has 1681 beams with 33 samples each, given 55,473 iterations for each emitter/receiver signal, the second VOI (Figure 22) has 451 beams with 255 samples each, given 101,475 iterations for each emitter/receiver pair. Thus, a ratio of 1.83 iterations to compute the beamforming in the second VOI, concerning the first VOI, can justify the slower time to make images in the second VOI.

The experimental 3-D images showed the flexibility achieved with the methodology developed in this work. In the first experiment the targets were imaged faster, even with a wider field of view, but at the cost of a narrow range. In the second experiment the targets were placed six times further than in the first experiment, but the images were obtained 1.8 times slower, even with a 75% smaller elevation range. For underwater applications, the first VOI can be adjusted to make faster and more detailed images of closer objects, widening the field of view to obtain more information about it. On the other hand, the second VOI can be useful for target detection missions at greater distances, restricting the view in the vertical direction while enlarging it in the horizontal direction. In the latter case, the greater the distance to detect a target, the greater the time to take an action, such as changing the navigation track or just reducing the navigation speed. Thus, the slower beamforming can be compensated. In addition, the two beamforming methods implemented using the 2-D array prototype make it possible to increase the quality of the images, at a greater computational cost.

## 6. Conclusions

A 2-D array ultrasonic transducer made of 1–3 piezocomposite elements was designed, constructed, and characterized. Acting as active sonar, by independently emitting and receiving ultrasonic pulses, the transducer can be used for 3-D imaging targets immersed in water.

To increase the bandwidth and the amplitude of the echoes, a composite matching layer made of alumina powder embedded in epoxy resin was added to the face of the transducer to match the acoustical impedance of the 1–3 piezocomposite to that of the water.

Furthermore, the series inductors match the electrical impedance of the elements with the transmitter/receptor system by nulling the reactive impedances near resonance frequencies. As a result, the magnitudes of the electrical impedances of the elements were reduced about ten times near the operational frequency of the transducer.

The characterization of transducers T1 (backing layer of air) and T2 (backing layer of epoxy resin) showed that an air backing layer can improve the sensitivity of the transducer, increasing the amplitude of the echoes up to 35% more than an epoxy resin backing. This was expected, because the transmission coefficient from the 1–3 piezocomposite to the air is almost zero, whereas that from the 1–3 piezocomposite to the epoxy resin is 28% (Table 2).

On the other hand, the bandwidth was also slightly improved with the air backing (8% wider than the bandwidth achieved using the epoxy resin backing). In general, the higher the emission, the lower the bandwidth. The difference between these two backing layers is not so relevant when a frontal matching layer and a 1–3 piezocomposite are used in the 2-D array. Also, this could have occurred because the excitation was a single half-wavelength square pulse. Therefore, a burst or CHIRP excitation could be considered to characterize new transducers in future works.

The acoustic field simulations showed the importance of improving the bandwidth, for reducing the loss of acoustic energy from the main lobe, whereas mitigating the grating lobes. Furthermore, the software implemented allows simulating a 3-D focused field.

The sign coherence factor was effective in the reduction of grating lobes noise. It allows the use of a pitch greater than half the wavelength in the propagating medium, leading to an increase in the angular resolution of the transducer.

The greater issue that limited the time to make each 3-D image was the computational load of the beamforming equation, which was implemented via the software Matlab^®^. Dynamic focusing is an iterative process that requires extremely high computational processing capacity.

Experimental 3-D images of plane objects immersed in water placed 0.2 m up to 1.15 m away from the transducer were made to validate the transducer. The transducer prototype design described here can be followed for the construction of other 2-D arrays for underwater applications.

Future works will be carried out with CHIRP excitations in an attempt to increase the amplitude of the echoes, whereas maintaining the wide bandwidth. A sparse 2-D array, with more elements, made from a 1–3 piezocomposite is indicated to increase the lateral angular resolution, allowing images of more complex scenarios.

## Figures and Tables

**Table 1:** Parameters used for acoustic field simulations.

Medium (Water)		Transducer of Square Elements				Sampling	
Density	Prop. velocity	M × N	Element size	Pitch	*f*_o_	Freq.	Δx,Δy,Δz
1000 kg/m^3^	1500 m/s	4 × 4	5 mm	5.2 mm	500 kHz	50 MHz	1 mm

**Table 2:** Properties and coefficients of the backing layers calculated relatives to the 1–3 piezocomposite element.

Transducer	Backing Layer	Propagation Velocity	Acoustical Impedance	*T_p_*	*R_p_*	*T_i_*	*R_i_*
T1	Air	340 m/s	≈400 rayl	≈0	−1	0	1
T2	Resin AW106/Hardener HV953U	2300 m/s	2.5 Mrayl	0.28	−0.72	0.49	0.51

**Table 3:** Characteristics measured with echoes from transducers T1 and T2 excited with a single rectangular negative pulse.

Transducer	Peak-to-Peak Voltage (V)	Bandwidth @ −6 dB (%)	Central Frequency (kHz)	Range Resolution (mm)	Angular Resolution Calculated @ *f_o_* (^o^)
T1 (Air Backing)	2.16 ± 0.36	54 ± 1	479 ± 5	2.94 ± 0.06	8.71 ± 0.09
T2 (Resin Backing)	1.60 ± 0.26	50 ± 2	480 ± 6	3.11 ± 0.11	8.69 ± 0.10
